# Patient-reported outcome measures after minimally invasive mitral valve surgery: The benefit may be early

**DOI:** 10.1016/j.xjon.2024.05.010

**Published:** 2024-05-27

**Authors:** Amy Brown, Rhys I. Beaudry, Jolene Moen, Sean Kang, Ali Fatehi Hassanabad, Vishnu Vasanthan, Alexander J. Gregory, William D.T. Kent, Corey Adams

**Affiliations:** Department of Cardiac Sciences, Libin Cardiovascular Institute, University of Calgary, Calgary, Alberta, Canada

**Figure:**
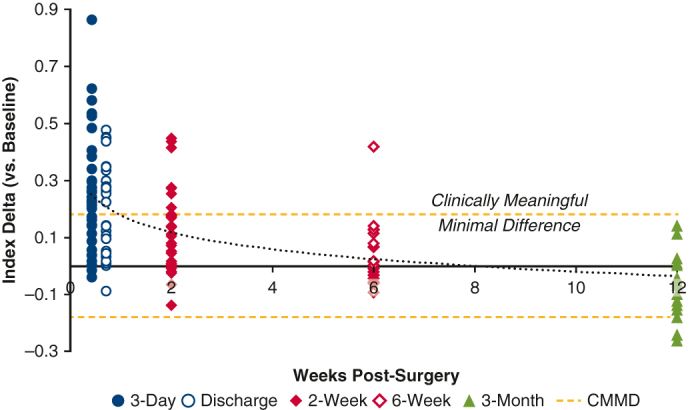
Change in EQ-5D Index score from baseline over time showing progressive patient recovery.

**Central Message**

Using EQ-5D-5L scores, most patients returned to baseline quality of life by 2 weeks after minimally invasive mitral valve surgery.

Minimally invasive cardiac surgery is associated with reduced pain, blood loss, transfusions, and hospital length of stay, compared with full median sternotomy (FMS).^1^^,^^2^ Along with conventional clinical outcomes, patient-reported outcome measures (PROMs) are necessary to inform a patient-centered decision-making model by quantifying patient perspectives.^3^

PROMs provide a structured approach to define physical, mental, and emotional components of the patient experience and can help determine health-related quality of life (QoL).^1^^,^^4^ The UK Mini Mitral Trial assessed physical functioning and return to usual activities at postoperative week 12 in patients randomized to minimally invasive mitral valve surgery (mini-MVS) or FMS-mitral valve surgery (FMS-MVS). The results showed no difference in mean change in physical function from baseline to 12 weeks, but benefits of minimally invasive approaches may become apparent at earlier time points.^5^ Observational reports suggest that the benefit of mini-MVS occurs earlier than 12 weeks.^1^^,^^4^ This prospective study aimed to assess health-related QoL defined by PROMs in the early postoperative period for patients who underwent mini-MVS.

## Methods

A single-center, prospective cohort study was performed for consenting patients receiving isolated mini-MVS. This study was approved by the Conjoint Health Research Ethics Board at the University of Calgary and conducted in accordance with the Declaration of Helsinki (Research Ethics Board identification: 20-0859; approved July 10, 2020).

Mini-MVS was performed through a right minithoracotomy with femoral cardiopulmonary bypass by 1 of 2 surgeons. Multimodal pain control was offered at each stage, including regularly scheduled acetaminophen and nonsteroidal anti-inflammatory drug supplementation, with opioids reserved for severe pain.

Along with operative and postoperative clinical outcomes, the 5-level EQ-5D (EuroQol) was used to collect health-related QoL at baseline, 3-days postoperative, discharge, and at 2, 6, and 12 weeks. The 5-level EQ-5D is composed of Likert scale questions for mobility, self-care, usual activities, pain/discomfort, and anxiety/depression and a visual analog scale (range, 0-100) for overall health. Return to baseline was determined as the first time point that the score matched or improved beyond the baseline score; data were censored thereafter. In the case that the QoL nadir occurred after hospital discharge, return to baseline was determined on the latter half of a patient’s recovery curve. The clinically meaningful minimal difference was calculated from the Likert scale index score, as previously reported, to represent the smallest amount of benefit that the patient can recognize and value.^3^

## Results

The study included 37 patients with postoperative follow-up to 12 weeks. Patient baseline characteristics, operative characteristics, and postoperative outcomes are shown in Table 1.

**Table 1:** Characteristics and outcomes of the included cohort (N = 37)

Variable	Result
Baseline	
Age (y)	62 ± 13
Male sex	19 (51)
Caucasian ethnicity	32 (86)
Body mass index	27.1 ± 4.1
Hypertension	9 (24)
Coronary artery disease	8 (22)
Diabetes	2 (5)
Smoking	1 (3)
COPD	1 (3)
Previous stroke or TIA	6 (16)
Atrial fibrillation	7 (19)
LVEF ≥60%	33 (89)
Operative	
Mitral valve repair	31 (84)
Cardiopulmonary bypass time (min)	118 ± 36
Aortic crossclamp time (min)	87 ± 31
Postoperative mitral regurgitation ≥ mild	5 (14)
Intensive care (d)	1.4 ± 1.4
Postoperative	
Atrial fibrillation	7 (19)
Stoke	1 (3)
Permanent pacemaker insertion	3 (8)
Transfusion	5 (14)
Acute kidney injury	1 (3)
Wound infection	0
Mortality	0
Total hospital length of stay (d)	7 ± 10

Values are presented as mean ± SD or n (%). *COPD*, Chronic obstructive pulmonary disease; *TIA*, transient ischemic attack; *LVEF*, left ventricular ejection fraction.

**Figure 1:**
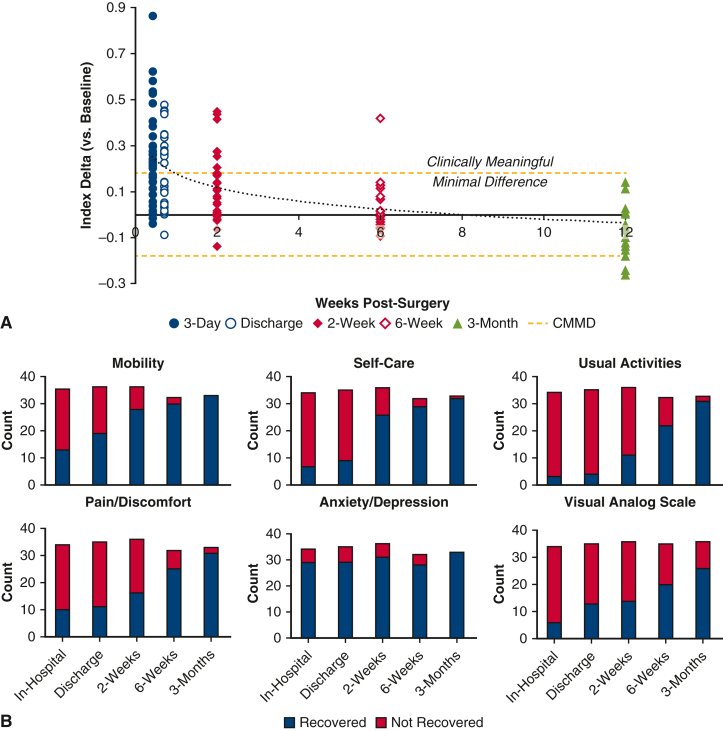
A, Change in EuroQol-5D Index score from baseline. Points above the clinically meaningful minimal difference line represent nonrecovered patients. Points below the clinically meaningful minimal difference line are recovered. The dotted line represents the mean change over time. B, Return to baseline (% recovered) for components of the 5-level EuroQol-5D, and EuroQol-5D vertical visual analog scale. *CMMD*, Clinically meaningful minimal difference.

At 2 weeks postsurgery, 81% of patients reported QoL similar to baseline, whereas the remaining 19% (n = 7) reported QoL deficits in excess of the suggested clinically meaningful minimal difference (Figure 1, *A*). There appeared to be variation in recovery time; however, all patients showed progressive recovery with 100% of patients returning to within the clinically insignificant deficit range by 12 weeks’ follow-up, indicating recovery (Figure 1, *A*).

All of the individual components of the 5-level EQ-5D showed progressive recovery over time (Figure 1, *B*). Notably, at 2 weeks postoperation, 78% of patients reported that their mobility returned to baseline, whereas only 31% of patients had returned to their baseline usual activities. By 6 weeks postoperative, 69% of patients had returned to baseline usual activities.

## Discussion

This study demonstrated that most patients returned to baseline overall QoL by 2 weeks after mini-MVS (Figure 1, *A* and *B*). This time point is earlier than studies have previously captured and suggests the potential for detecting differences between mini-MVS and FMS-MVS before complete recovery at 12 weeks postoperative.^4^^,^^5^

A major aim of mini-MVS is to promote faster return to baseline compared with the FMS-MVS approach. In fact, patients have reported that the most important deciding factor between surgical approaches is the timing of return to physical function after surgery.^5^ The UK Mini Mitral Trial showed a nonsignificant difference in physical functioning at 12 weeks between mini-MVS and FMS-MVS; however, subtle differences between approaches may have been washed out by nearly complete recovery at 12 weeks.^5^ Similarly, our results demonstrated that all patients had returned to baseline QoL by 12 weeks. It is important to consider how quickly patients return to their presurgery baseline to inform patient-centered decision making. Currently, evidence shows return to baseline at 12 weeks regardless of the surgical approach, but more data are needed to better understand whether meaningful differences exist earlier in convalescence.^1^^,^^5^

## Conclusions

PROMs represent outcomes important to patients and should be used as tools for patient-centered decision making. QoL PROMs appear to be equivocal between FMS-MVS and mini-MVS at 12 weeks, but the earlier postoperative period should not be disregarded. Future studies comparing FMS-MVS to mini-MVS should report PROMs before 12 weeks postoperative.

## Conflict of Interest Statement

The authors reported no conflicts of interest.

The *Journal* policy requires editors and reviewers to disclose conflicts of interest and to decline handling or reviewing manuscripts for which they may have a conflict of interest. The editors and reviewers of this article have no conflicts of interest.
